# The most unusual diplostomoideans from atypical hosts: *Pipatrema mirabilis* n. sp., n. g. (Digenea: Diplostomidae) from *Pipa pipa* (Amphibia: Pipidae) in Brazil and a related species from fish in Argentina

**DOI:** 10.1016/j.crpvbd.2026.100369

**Published:** 2026-03-26

**Authors:** Vasyl V. Tkach, Tyler J. Achatz, Martin M. Montes, Jorge Barneche, Bianca Nandyara, Francisco Tiago de Vasconcelos Melo

**Affiliations:** a Department of Biology, University of North Dakota, Starcher Hall, 10 Cornell Street Stop 9019, Grand Forks, ND, 58202, USA; b Department of Natural Sciences, Middle Georgia State University, Macon, GA, 31206, USA; c Centro de Estudios Parasitológicos y Vectores (CEPAVE), Consejo Nacional de Investigaciones Científicas y Técnicas, Universidad Nacional de La Plata (CCT, CONICET-UNLP), La Plata, Buenos Aires, Argentina; d Laboratory of Cell Biology and Helminthology “Prof. Dr. Reinalda Marisa Lanfredi”, Institute of Biological Sciences, Federal University of Para, Belem, PA, Brazil

**Keywords:** Diplostomoidea, New genus, New species, *Pipatrema mirabilis*, *Pipa pipa*, Brazil, Molecular phylogeny

## Abstract

The Superfamily Diplostomoidea is a major lineage of digenean trematodes characterized by the presence of a ventral holdfast organ. Its members parasitize the intestines of tetrapod vertebrates worldwide, with only a single genus reported from fishes and none from amphibians. During a biodiversity survey in the Brazilian Amazon (Pará State), we discovered highly unusual diplostomoidean digeneans lacking a holdfast organ in the fully aquatic anuran *Pipa pipa* (Pipidae). We describe these digeneans as a new species and genus *Pipatrema mirabilis* n. sp., n. g. Additionally, a single specimen of the same genus was found in *P. pipa* from Amapá State, Brazil and a single specimen belonging to a related genus and species was collected from the South American catfish *Rhamdia quelen* (Siluriformes: Heptapteridae) in Argentina. The molecular phylogeny based on the 28S rRNA gene revealed a secondary loss of the holdfast organ, a defining morphological feature of diplostomoideans. Furthermore, the phylogenetic analysis positioned both taxa lacking a holdfast organ within the proterodiplostome clade among digeneans parasitic in South American crocodilians. These results strongly suggest that parasitism in fish and amphibians is the result of secondary evolutionary host-switching events. This study provides the first record of adult diplostomid trematodes from amphibian and fish hosts. Our findings highlight the importance of integrative approaches combining morphology, molecular phylogenetics, and ecological context to understand parasite diversification and host-parasite evolution in complex aquatic systems.

## Highlights

• First diplostomoideans without a holdfast organ are reported.
• A new genus and species is described from *Pipa pipa* in Brazil.
• This is the first report of adult diplostomids from fishes and amphibians.
• Molecular phylogeny suggests secondary loss of the hodfast organ.
• Transition from reptiles to fishes and amphibians is a secondary host-switching event.

## Graphical abstract

**Figure:**
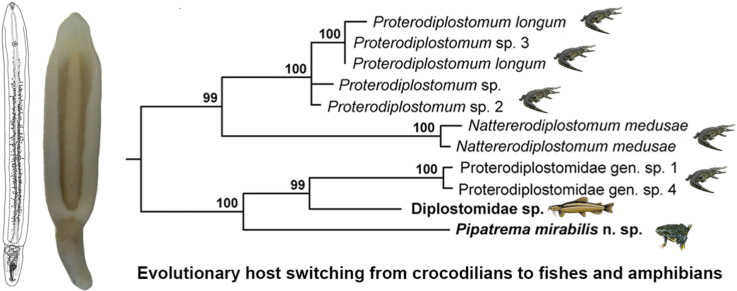

## 1 Introduction

The Diplostomoidea Poirier, 1886 is one of the major basal lineages of digenean trematodes that are parasitic as adults in tetrapod vertebrates worldwide, with only a single genus reported from fishes and none from amphibians (Niewiadomska, 2002; Olson et al., 2003; Achatz et al., 2019; Achatz and Tkach, 2025). According to the latest major treatment of the entire superfamily in the “Keys to the Trematoda”, the most defining feature of the superfamily Diplostomoidea is the “Body with specialized holdfast organ situated posterior to ventral sucker” (Niewiadomska, 2002). Accordingly, all diplostomoidean taxa described thus far had a stronger or weaker developed holdfast organ situated on the ventral side of their prosoma. One of the major monophyletic diplostomoidean lineages, formerly the Proterodiplostomidae Dubois, 1936 (recently synonymized with the Diplostomidae by Achatz and Tkach, 2025), has yet another unique morphological characteristic, namely the paraprostate associated with the terminal parts of their reproductive system at the posterior end of the body. Until now, fully developed adult members of this lineage were reported from reptilian hosts only, with majority of its members parasitic in crocodilians and a few taxa known from other reptiles such as turtles and snakes (Niewiadomska, 2002; Tellez, 2013; Tkach et al., 2020; Achatz et al., 2021, 2022; Achatz and Tkach, 2025).

During a biodiversity study of vertebrates and their parasites in the Brazilian Amazon, we have discovered highly unusual diplostomoidean digeneans possessing paraprostate but lacking a holdfast organ, in a unique, fully aquatic anuran amphibian, the common Surinam toad *Pipa pipa* L. (Pipidae). Detailed analysis has demonstrated that they belong to a new species and new genus of diplostomoideans. Herein, we describe this new species morphologically, differentiate it from known diplostomoidean taxa, and analyze its phylogenetic affinities based on sequences of the large subunit of nuclear ribosomal RNA gene (28S rDNA). A partial sequence of mitochondrial cytochrome *c* oxidase subunit 1 (*cox*1) was generated for future comparisons. Additionally, an adult specimen of another related species was collected from the South American catfish (or bagre) *Rhambdia quelen* (Quoy & Gaimard) (Siluriformes: Heptapteridae) in Argentina and used for molecular analysis.

## 2 Materials and methods

### 2.1 Sample collection and morphological study

Eight adult diplosomids lacking a holdfast organ were found in the intestine of a single examined *P. pipa* (Anura: Pipidae) from the Caxiuanã National Forest (Floresta Nacional de Caxiuanã) in Pará State, Brazil (1°47′32.3″S, 51°26′02.5″W) (license numbers: SISBIO 64103-1 and SISBIO 67714-1). A morphologically very similar single specimen of diplostomid was collected in March 2018 from the intestine of *P. pipa* from Santana, Amapá State, Brazil (0°02′11.34"S, 51°09′39.14"W) (license permission SISBIO 53557-8). Additionally, a single adult specimen of a proterodiplostomid digenean was obtained from the intestine of a bagre (*R*. *quelen*) collected in Buñirigo Stream, Magdalena District, Buenos Aires Province, Argentina (35°03′46″S, 57°33′15″W).

Live digeneans were rinsed in saline, heat-killed with hot water and immediately fixed in 70% ethanol. For morphological analysis, 7 digeneans of the new species from *P. pipa* were stained with aqueous alum carmine following the protocol provided by Lutz et al. (2017) and studied using a Leica DMC 4500 microscope (Buffalo Grove, Illinois, USA) equipped with DIC, digital imaging/measuring system and a drawing tube. Pencil drawings were made with the aid of a drawing tube; publication-quality figures were prepared using Adobe Illsutrator software. All measurements are in micrometres. The type-series was deposited in the collection of the Museu Paraense Emílio Goeldi, Belém, Brazil (MPEG) and the hologenophore of the specimen collected in Argentina from *R. quelen* was deposited in the collection of Museo de Ciencias Naturales de La Plata (MLP-He 8348).

### 2.2 Molecular study

**Supplementary Fig. S1:**
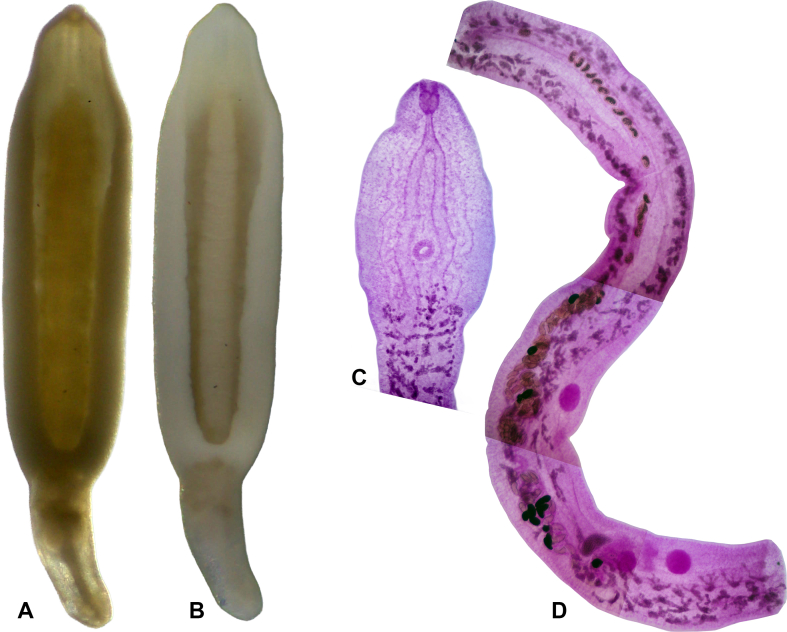
**A, B**, Specimen from *Pipa pipa* collected in Santana, Amapá State, Brazil. **C**, **D**, Fragments of the hologenophore of the specimen collected from *R. quelen* in Magdalena District, Buenos Aires Province, Argentina.

Due to the scarcity of material, we extracted genomic DNA from a single specimen of the new species from *P. pipa* and also used the mid-section of the only available specimen of the diplostomid from *R. quelen*, for extraction following a protocol provided by Tkach and Pawlowski (1999). The remaining fragments of the specimen from *R. quelen* were stained with carmine and mounted (Supplementary Fig. S1C and D). All attempts to obtain good PCR products from the extracted fragment of the single specimen from *P. pipa* collected in Santana were unsuccessful. The specimen was photographed in ethanol prior to excision of a small piece for extraction (Supplementary Fig. S1A and B). The remainder of the specimen was kept for a future attempt to obtain DNA using DNA enrichment methods. We amplified fragments of 28S rRNA and mitochondrial cytochrome *c* oxidase subunit 1 (*cox*1) genes using polymerase chain reactions (PCR). The PCR amplifications of 28S were performed using the forward primer digL2 (5′-AAG CAT ATC ACT AAG CGG-3′) and the reverse primer 1500R (5′-GCT ATC CTG AGG GAA ACT TCG-3′) (Tkach et al., 2003). The fragment of the *cox*1 gene of the specimen from *P. pipa* was amplified using the forward primer Dipl_Cox_5’ (5′-ACK TTR GAW CAT AAG CG-3′) and the reverse primer Dipl650R (5′-CCA AAR AAY CAR AAY AWR TGY TG-3′) (Achatz et al., 2021). The *cox*1 fragment of the specimen from *R. quelen* was amplified using the forward primer DICE1F (5′-TTW CNT TRG ATC ATA AG-3′) and the reverse primer DICE14R (5′-CCH ACM RTA AAC ATA TGA TG-3′) (Van Steenkiste et al., 2015). PCR amplifications were carried out in a total volume of 25 μl using One-Taq quick load PCR mix from New England Biolabs (Ipswich, Massachusetts, USA) according to the manufacturer’s instructions. Annealing temperatures of 53 °C and 45 °C were used for the nuclear ribosomal and mitochondrial gene amplifications, respectively.

Illustra ExoProStar PCR clean-up enzymatic kit from Cytiva (Marlborough, Massachusetts, USA) was used to purify PCR products and BrightDye Terminator Cycle Sequencing Kit (MCLAB, California, USA) was used to cycle-sequence purified PCR products. Sequencing reactions were cleaned using a BigDye Sequencing Clean Up Kit from MCLAB and subsequently run on an ABI 3130 automated capillary sequencer (Thermo Fisher Scientific, Waltham, Massachusetts, USA). The PCR primers were used for sequencing reactions of both gene fragments. In addition, internal forward primer DPL600F (5′-CGG AGT GGT CAC CAC GAC CG-3′) and internal reverse primer DPL700R (5′-CAG CTG ATT ACA CCC AAA G-3′) were used for sequencing of 28S amplicons (Achatz et al., 2019, 2021). Contiguous sequences were assembled using Sequencher 4.2 software (GeneCodes Corp., Ann Arbor, Michigan, USA). All newly generated sequences are deposited in GenBank under the accession numbers PZ174004-PZ174005 (28S rDNA) and PZ172967-PZ172968 (*cox*1).

### 2.3 Phylogenetic analyses

To reveal the phylogenetic position of the new species within the Diplostomidae, the newly obtained 28S rDNA sequences of the new species and the specimen from fish were aligned with 73 previously published 28S rDNA sequences representing all main lineages of diplostomoideans and the sequence of a cyathocotyid, *Suchocyathocotyle crocodili* (GenBank: MK650450), used as an outgroup. The outgroup was chosen based on the phylogenetic analysis of Achatz and Tkach (2025). Based on the result of the phylogenetic analysis using the broad selection of diplostomoideans, a smaller second alignment was prepared that included the newly obtained sequences of the new species and the specimen from fish with 33 previously published 28S rDNA sequences of the proterodiplostomid clade and *Alaria mustelae* Bosma, 1931, used as the outgroup. Sequences were aligned using ClustalW implemented in MEGA 7 (Kumar et al., 2016) with default parameters. The alignments were trimmed to the length of the shortest sequence; nucleotide positions with ambiguous homology were excluded from the analyses.

The phylogenetic analysis was conducted using Bayesian inference as implemented in MrBayes v3.2.6 software (Ronquist and Huelsenbeck, 2003). The general time-reversible model with estimates of invariant sites and gamma-distributed among-site variation (GTR+G+I) model was identified as the best-fitting nucleotide substitution model for both alignments using MEGA 7 (Kumar et al., 2016). The phylogenetic analysis was performed using MrBayes software as follows: Markov chain Monte Carlo (MCMC) chains were run for 6,000,000 generations with sampling frequency set at 1000. The number of generations was determined to be sufficient because the standard deviation stabilized below 0.01. Log-likelihood scores were plotted and only the final 75% of trees were retained to produce the consensus trees.

## 3 Results

### 3.1 Descriptions of new taxa

#### 3.1.1 *Pipatrema* Tkach, Achatz & Melo n. g.

*Diagnosis*: Body distinctly bipartite; prosoma extremely elongate, 6–8 times longer than very short opisthosoma; margins of prosoma elevated almost the entire length of prosoma. Oral sucker small, subterminal; ventral sucker small, similar in size to oral sucker, at the border of anterior 1/5–1/6 of prosoma. Pseudosuckers absent. Holdfast organ absent. Prepharynx absent, pharynx very small, esophagus very short, similar in length to pharynx; ceca running close to each other the whole length of prosoma and run laterally in opisthosoma, almost reaching posterior end of body. Testes 2, tandem. Seminal vesicle convoluted, ejaculatory duct nearly straight, starting approximately at level of end of ceca. Paraprostate elongated, surrounded by glandular cells, dorsal to distal ducts of male and female reproductive systems. Ovary pretesticular; oötype intertesticular. Vitellarium consisting of small vitelline follicles concentrated closely along ceca from level just posterior to oral sucker to end of prosoma. Uterus short, first goes anteriorly, then turns posteriorly at border between prosoma and opisthosoma; eggs few. Metraterm, ejaculatory duct and paraprostate open into short hermaphroditic duct which opens into shallow genital atrium. Genital atrium with subterminal opening on dorsal side. Excretory pore terminal. In tongueless frogs (Pipidae). South America.

*Type-species*: *Pipatrema mirabilis* n. sp.

*Remarks*: *Pipatrema* n. g. can be easily distinguished from all other diplostomid and diplostomoidean genera by the complete lack of a holdfast organ. It also differs from all other adult diplostomoideans with distinct opisthosoma by the unusually long prosoma with prosoma:opisthosoma length ratio of 6–8. Although our specimen from *R. quelen* in Argentina is phylogenetically related to the new species (see below), it is dramatically different morphologically in many characteristics, most importantly in the prosoma:opisthosoma length ratio, as well as relative position of gonads and their arrangement in opisthosoma (Supplementary Fig. S1C and D). Our single specimen from *P. pipa* in Santana is rather similar to the new species but has a relatively shorter prosoma (Supplementary Fig. S1A and B). Unfortunately, we cannot comment more on that specimen as it is still being used in an attempt to obtain sequencable DNA from it.

#### 3.1.2 *Pipatrema mirabilis* Tkach, Achatz & Melo n. sp.

##### 3.1.2.1 Taxonomic summary

*Type-host*: *Pipa pipa* (Linnaeus) (Anura: Pipidae Gray).

*Type-locality*: Caxiuanã National Forest (Floresta Nacional de Caxiuanã), Melgaço and Portel municipalities, Pará State, Brazil (1°47′32.3″S, 51°26′02.5″W).

*Type-material*: Holotype: MPEG 391, labeled ex. *Pipa pipa*, small intestine, Caxiuanã National Forest (Floresta Nacional de Caxiuanã), Melgaço and Portel municipalities, Pará State, Brazil, 11 Nov 2015, coll. F.T.V. Melo. Paratypes: MPEG 392–397 (lot of 6 slides), labels identical to the holotype.

*Site in host*: Small intestine.

*Representative DNA sequences*: PZ174004 (28S rDNA), PZ172967 (*cox*1).

*ZooBank registration*: To comply with the regulations set out in Article 8.5 of the amended 2012 version of the International Code of Zoological Nomenclature (ICZN, 2012), details of the new species and the new genus have been submitted to ZooBank. The Life Science Identifier (LSID) of the article is urn:lsid:zoobank.org:pub:C7BFF289-6B91-434C-9261-9218720EEAFD. The LSIDs for the new names are as follows: *Pipatrema* Tkach, Achatz & Melo n.g. (urn:lsid:zoobank.org:act:49C7E35B-1FA4-4564-B1BF-82271E4F2B04); and *Pipatrema mirabilis* Tkach, Achatz & Melo n. sp. (urn:lsid:zoobank.org:act:8E405E03-341C-4131-B4FC-EE3A50F02607).

*Etymology*: The name of the new genus reflects the genus name of its amphibian host. The specific epithet refers to the unique morphology of the new species.

##### 3.1.2.2 Description

**Fig. 1:**
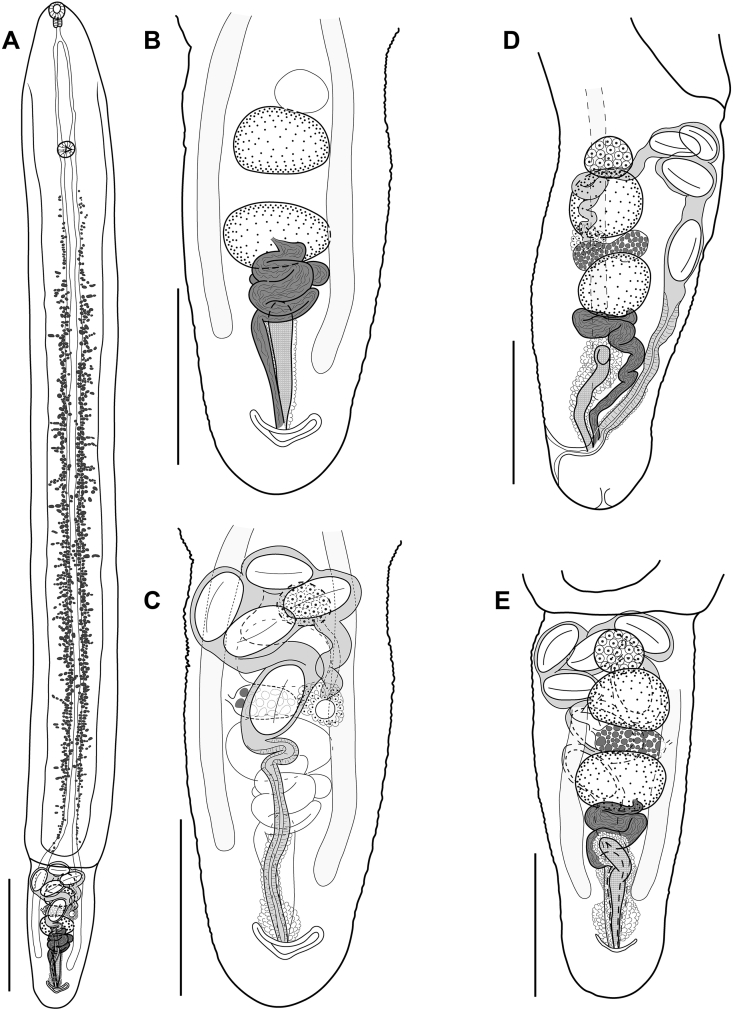
*Pipatrema mirabilis* n. sp. **A** Holotype, total view, ventral. **B**, **C** Paratype, opisthosoma, showing different parts of the reproductive system, dorsal view. **D** Paratype, opisthosoma, dextral lateral view. **E** Paratype, opisthosoma, dorsal view.

[Based on 6 adult specimens; a single laterally positioned specimen was not measured; measurements of the holotype are given in text; measurements of the entire series are provided in Table 1; Fig. 1.] Body distinctly bipartite; prosoma 3688 × 390, extremely elongate, 6 times longer than opisthosoma, 619 × 296. Prosoma with nearly parallel margins elevated almost the entire length of prosoma (Fig. 1). Opisthosoma widest right after the junction between prosoma and opisthosoma. Prosoma:opisthosoma length ratio 6.0. Oral sucker small, subterminal, 73 × 77; ventral sucker small, similar in size to oral sucker (identical in holotype), 71 × 76, at border of anterior 1/5−1/6 of prosoma. Forebody 645 (15% of total body length). Pseudosuckers absent. Holdfast organ absent. Pre-pharynx absent, pharynx very small, 50−45; oral sucker:pharynx width ratio 1.7. Esophagus very short, 55, similar in length to pharynx; ceca running very close to each other along whole length of prosoma and more laterally on either side of opisthosoma, almost reaching posterior end of body, terminating 174 from it.

**Table 1:** Morphometric data for *Pipatrema mirabilis* n. sp.

Character	Range (Mean)
Body length	4307–6345 (5221)
Prosoma length	3688–5626 (4560)
Prosoma width	348–437 (388)
Opisthosoma length	619–719 (661)
Opisthosoma width	249–339 (294)
Prosoma:opisthosoma length ratio	5.9–7.9 (6.9)
Forebody length	523–680 (615)
Forebody length as % of body length	10.6–15.0 (11.9)
Oral sucker length	72–89 (77)
Oral sucker width	70–94 (80)
Pharynx length	48–55 (51)
Pharynx width	45–58 (50)
Oral sucker:pharynx width	1.5–1.8 (1.6)
Esophagus length	40–77 (58)
End of ceca to posterior end	164–191 (177)
Ventral sucker length	55–89 (70)
Ventral sucker width	64–104 (108)
Oral sucker:ventral sucker width ratio	0.83–1.11 (0.97)
Anterior testis length	90–124 (108)
Anterior testis width	133–168 (148)
Posterior testis length	91–130 (110)
Posterior testis width	134–171 (155)
Distance between testes	26–40 (35)
Paraprostate length	141–202 (181)
Paraprostate width	40–49 (43)
Ovary length	59–89 (74)
Ovary width	74–95 (86)
Egg number	5–9 (7)
Egg length	98–114 (106)
Egg width	49–65 (60)

Testes 2, tandem, intercecal, rounded or transversely elongate, in middle portion of opisthosoma; anterior testis 90 × 136, posterior testis 91 × 150. Seminal vesicle convoluted, ejaculatory duct nearly straight, starting approximately at level of end of ceca. Paraprostate elongated, 177 × 40, surrounded by glandular cells, dorsal to distal ducts of male and female reproductive systems.

Ovary 59 × 74, pretesticular; oötype intertesticular. Vitellarium consisting of long bands of small vitelline follicles in prosoma arranged closely along ceca from level just posterior to oral sucker to end of prosoma. Vitelline reservoir 47 × 92, situated between testes. Uterus short, first going anteriorly, then turning posteriorly at border between prosoma and opisthosoma. Uterus contains 5–9 eggs (5 in holotype); eggs, 104−108 × 58−62. Metraterm, male duct and paraprostate open into short hermaphroditic duct which opens into shallow genital atrium. Genital atrium with subterminal opening on dorsal side.

Excretory pore terminal.

##### 3.1.2.3 Remarks

See the remarks after the diagnosis of the new genus above.

### 3.2 Molecular phylogeny

**Fig. 2:**
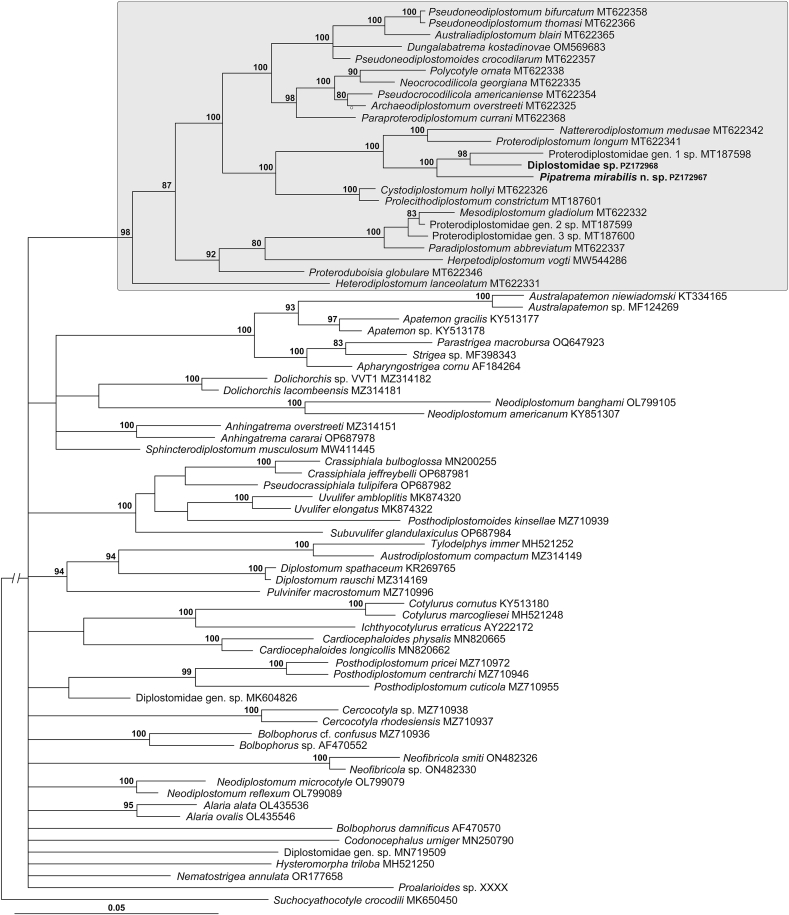
Phylogenetic interrelationships among 75 diplostomid taxa based on Bayesian Inference (BI) analysis of partial 28S rRNA gene sequences. Bayesian inference posterior probability values lower than 80% are not shown. The new sequences generated in this study are indicated in bold. The proterodiplostome clade is indicated by a shaded rectangle. The scale-bar indicates the number of substitutions per site.

**Fig. 3:**
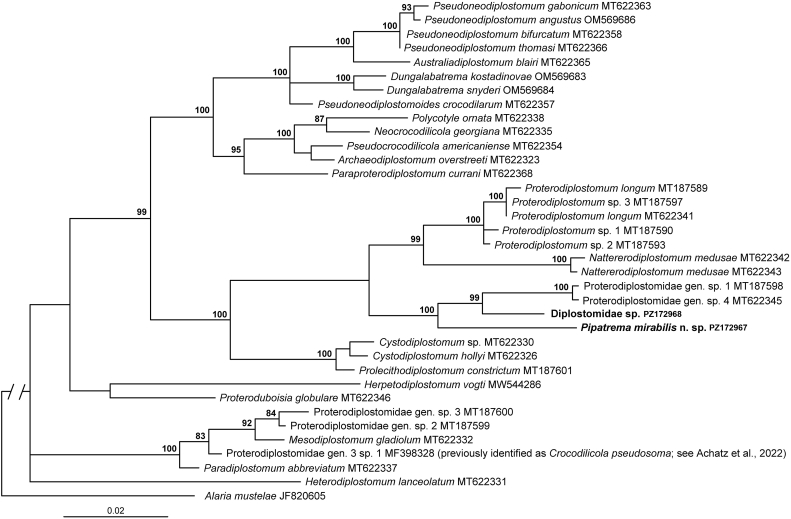
Phylogenetic interrelationships among 35 taxa belonging to the proterodiplostome clade based on Bayesian Inference (BI) analysis of partial 28S rRNA gene sequences. Bayesian inference posterior probability values lower than 80% are not shown. The new sequences generated in this study are indicated in bold. The scale-bar indicates the number of substitutions per site.

The phylogenetic tree resulting from the analysis of the broader 28S rDNA dataset was characterized by extensive basal polytomy and well-resolved main clades (Fig. 2). The branch topology and branch support values were similar to those reported by Achatz and Tkach (2025). The new species from *P. pipa* and the digenean from fish in Argentina appeared in the 98% supported major clade of taxa formerly belonging to the family Proterodiplostomidae, therefore we conducted the second anaysis which included a greater diversity of taxa of the proterodiplostome lineage (Fig. 3).

In this second phylogenetic tree, our digeneans from *P. pipa* and fish were placed in a 100% supported clade where all remaining taxa were represented by parasites of crocodilians. One sub-clade included members of *Prolecithodiplostomum* and *Cystodiplostomum* while the other sub-clade split into two clusters: a 99% supported group of *Proterodiplostomum* spp. and *Nattererodiplostomum medusae*, and a 100% supported cluster uniting the new species from *P. pipa*, the species from fish in Argentina, and an unidentified species from *Caiman yacare* Daudin in Pantanal, Brazil (GenBank: MT187598) (Fig. 3).

## 4 Discussion

This is the first report of adult diplostomids from the intestine of fish and amphibians. Until the present study, no adult diplostomid digeneans were known from amphibians worldwide, although progenetic ovigerous metacercariae of *Proalarioides* Yamaguti, 1933 were reported from amphibians in India (for overview, see Achatz et al., 2024). Considering that adult diplostomids were also not known from fish hosts, we initially hypothesized that our new species might represent a basal lineage to other diplostomids, or at least to members of the proterodiplostome clade. However, contrary to our initial hypothesis, the phylogenetic results (Fig. 2, Fig. 3) demonstrated that our specimens from both the amphibian host (*P. pipa*) in Brazil and the fish host (*R. quelen*) in Argentina were found nested within the proterodiplostome clade consisting of several genera parasitic in South American crocodilians (caimans). Thus, parasitism in fishes and amphibians resulted from a secondary evolutionary host-switching event. Although such a transition undoubtedly required significant adaptation, it was likely facilitated by shared ecological characteristics of the hosts, including the habitat use and dietary overlap.

Although digeneans from *P. pipa* in Brazil and from *R. quelen* in Argentina were positioned in the same terminal cluster, they were not the closest sister groups. This, combined with significant morphological differences, clearly indicates that our specimens from anurans and fish belong to different genera.

The genus *Pipa* comprises seven species of predominantly or exclusively aquatic anuran amphibians of the family Pipidae (which also includes three genera of African clawed frogs) distributed across southern Central America and northern South America. Considering that their diet often includes small fishes and amphibians, including tadpoles (Gines, 1958; Trueb and Massemin, 2001), it is plausible that repeated consumption of hosts harboring metacercariae over a long period of time has resulted in the ancestors of *Pipatrema* gaining the ability to survive, develop and mature in the intestine of new hosts.

Besides, the transition from one group of ectothermic vertebrates (reptiles) to other ectothermic vertebrates (fishes and amphibians) was likely less physiologically demanding than, for instance, a jump between endothermic and ectothermic vertebrates. Furthermore, considering that these digeneans have been found only in South America, we hypothesize that this host switching has occurred relatively recently in the evolutionary history of diplostomids.

Additionally, the hydrological regimes of the Amazon and Paraná-Paraguay basins are governed by the flood pulse, an ecological force that promotes seasonal connectivity between terrestrial and aquatic habitats (Junk et al., 1989; Junk and Wantzen, 2004). We propose that this phenomenon acts as an evolutionary pressure, in which rising water levels expand the shared microhabitat among crocodilians, fishes, and pipid frogs. This seasonal dynamic aids significant spatial and dietary overlap, creating an ecological opportunity for the transmission of metacercariae. In the context of *Pipatrema*, the flood pulse likely catalyzed the host switching by allowing ancestral reptilian parasites to colonize new ectothermic hosts through repeated ingestion in these highly dynamic floodplain environments.

Likewise, the fact that our new species completely lacked the holdfast organ, which is present in all other diplostomoidean genera, also raised the question whether it may potentially be the basal taxon of the Diplostomoidea that never had a holdfast organ, or its absence is the result of a secondary evolutionary loss. Once again, molecular phylogeny (Fig. 2, Fig. 3) provided strong evidence that the lack of the holdfast organ in digeneans reported herein from an amphibian and a fish is the result of a secondary evolutionary event.

Host switching events based on dietary and spatial overlaps are not uncommon among parasites, including digeneans. For instance, such host switching within the same general regions (the Brazilian Amazon and the Brazilian Pantanal) has been recently demonstrated by Achatz et al. (2025) among members of the diplostomid genus *Neodiplostomum*. In the latter case, host switching was also accompanied by a significant morphological change in the form of unique structures at the anterior extremity. The discovery of two different but closely related species within the same clade in relatively distant geographic areas, i.e. the Brazilian Amazon and northern Argentina, in the present study suggests strong potential for the discovery of additional members of this diplostomid lineage from other regions and hosts across the continent. At the same time, given the unique lifestyle of *Pipa* spp., we expect that additional species can be found in other *Pipa* spp. and fishes rather than in members of other groups of amphibians.

## 5 Conclusion

Our findings reveal a previously unknown evolutionary trajectory within the Diplostomidae, documenting the first record of adult forms successfully colonizing amphibians and fish through a secondary host switch from crocodilians. This transition was accompanied by a remarkable morphological change, namely the secondary loss of the holdfast organ, thus highlighting the adaptive plasticity of even the most typical structures in digeneans during colonization of new hosts. The presence of this lineage in both the Amazon and the Paraná-Paraguay basins suggests that the diversification of these parasites is closely linked to the aquatic connectivity and predator-prey dynamics of South American wetlands. Future research should target other species of *Pipa* and syntopic fish to fully unravel the diversity of this specialized diplostomid clade.

## Ethical approval

All procedures contributing to this work comply with applicable institutional, national, and international guidelines for animal care and use, as approved by the Animal Research Ethics Committee of the Federal University of Pará, under license 8341260821. The present study was registered in the National System for the Management of Genetic Heritage and Associated Traditional Knowledge (SisGen) under record number A8626D1 in compliance with Brazilian Law No. 13,123/2015. Collecting of amphibians in Pará State and Amapá State, Brazil was conducted under permits No. SISBIO 64103-1, SISBIO 67714-1 and SISBIO 53557-8. Fish collecting in Argentina was conducted under permit DI-2019-49-GDEBA-DANPOPDS from the Ministerio de Medio Ambiente de la Provincia de Buenos Aires.

## CRediT authorship contribution statement

**Vasyl V. Tkach:** Conceptualization, Investigation, Data curation, Formal analysis, Funding acquisition, Methodology, Resources, Writing - original draft, Writing - review & editing. **Tyler J. Achatz:** Conceptualization, Data curation, Formal analysis, Methodology, Writing - original draft, Writing - review & editing. **Martin M. Montes:** Investigation, Data curation, Resources, Funding acquisition, Formal analysis, Writing - review & editing. **Jorge Barneche:** Investigation, Data curation, Writing – review & editing. **Bianca Nandyara:** Investigation, Data curation, Writing – review & editing. **Francisco Tiago de Vasconcelos Melo:** Investigation, Data curation, Resources, Funding acquisition, Formal analysis, Writing - review & editing.

## Funding

This study was supported by the National Science Foundation (grant DEB-1120734) and National Institutes of Health (grant R15AI092622) for Vasyl Tkach as well as the University System of Georgia Stem Initiative IV (Middle Georgia State University) for Tyler Achatz. This study was also supported by Coordination for the Improvement of Higher Education Personnel, Brazil (CAPES); the National Council for Scientific and Technological Development (CNPq) Research Productivity Scholarship to Francisco T.V. Melo (Process number 314116/2021–4), Amazon Foundation for Research and Studies Support (FAPESPA)/CNPq–PRONEM (01/2021 process number 794027/2013).

## Declaration of competing interest

The authors declare that they have no known competing financial interests or personal relationships that could have appeared to influence the work reported in this paper.

## Data Availability

All data generated or analyzed during this study are included in this published article and its supplementary file. All newly generated sequences are deposited in GenBank under the accession numbers PZ174004-PZ174005 (28S rDNA) and PZ172967-PZ172968 (*cox*1).

## References

[bib1] Achatz T.A., Chacko S., Prasadan P.K., Tkach V.V. (2024). Proterodiplostomid no longer: molecular phylogeny reveals the true position of *Proalarioides* (Digenea: Diplostomoidea). Parasitol. Int..

[bib3] Achatz T.J., Brito E.S., Fecchio A., Tkach V.V. (2021). Description and phylogenetic position of a new species of *Herpetodiplostomum* from *Phrynops geoffroanus* in Brazil and a re-evaluation of *Cheloniodiplostomum*. J. Parasitol..

[bib2] Achatz T.J., Kostadinova A., Georgieva S., Fecchio A., Tkach V.V. (2025). Host switching at water edge: phylogeny and systematics of diplostomids (Digenea: Diplostomidae) from passeriform and cuculiform birds in South America. Syst. Biodivers..

[bib4] Achatz T.J., Martens J.R., Kostadinova A., Pulis E.E., Orlofske S.A., Bell J.A. (2022). Molecular phylogeny of *Diplostomum, Tylodelphys, Austrodiplostomum* and *Paralaria* (Digenea: Diplostomidae) necessitates systematic changes and reveals a history of evolutionary host switching events. Int. J. Parasitol..

[bib5] Achatz T.J., Pulis E.E., Junker K., Binh T.T., Snyder S.D., Tkach V.V. (2019). Molecular phylogeny of the Cyathocotylidae (Digenea, Diplostomoidea) necessitates systematic changes and reveals a history of host and environment switches. Zool. Scr..

[bib6] Achatz T.J., Tkach V.V. (2025). Recent advances and current state of knowledge of phylogenetics and systematics of the Diplostomoidea with a proposal of a new classification system and a key to genera. J. Helminthol..

[bib7] Gines H. (1958). Representantes de la familia Pipidae (Amphibia: Salientia) en Venezuela. Mem. Soc. Cienc. Nat. La Salle.

[bib8] ICZN (2012). International Commission on Zoological Nomenclature: amendment of Articles 8, 9, 10, 21 and 78 of the International Code of Zoological Nomenclature to expand and refine methods of publication. Bull. Zool. Nomencl..

[bib9] Junk W.J., Bayley P.B., Sparks R.E. (1989). The flood pulse concept in river-floodplain systems. Can. Spec. Publ. Fish. Aquat. Sci..

[bib10] Junk W.J., Wantzen K.M., Welcomme R.L., Petr T. (2004). Proceedings of the Second International Symposium on the Management of Large Rivers for Fisheries, Bangkok.

[bib11] Kumar S., Stecher G., Tamura K. (2016). MEGA 7: Molecular Evolutionary Genetics Analysis version 7.0 for bigger datasets. Mol. Biol. Evol..

[bib12] Lutz H.L., Tkach V.V., Weckstein J.D., Webster M. (2017). The Role of Collections in Ornithology: the Extended Specimen. Studies in Avian Biology.

[bib13] Niewiadomska K., Gibson D.I., Jones A., Bray R.A. (2002).

[bib14] Olson P.D., Cribb T.H., Tkach V.V., Bray R.A., Littlewood D.T.J. (2003). Phylogeny and classification of the Digenea (Platyhelminthes: Trematoda). Int. J. Parasitol..

[bib15] Ronquist F., Huelsenbeck J.P. (2003). MRBAYES 3: Bayesian phylogenetic inference under mixed models. Bioinformatics.

[bib16] Tellez M. (2013).

[bib17] Tkach V.V., Achatz T.J., Pulis E.E., Junker K., Snyder S.D., Bell J.A. (2020). Phylogeny and systematics of the Proterodiplostomidae Dubois, 1936 (Digenea: Diplostomoidea) reflect the complex evolutionary history of the ancient digenean group. Syst. Parasitol..

[bib18] Tkach V.V., Littlewood D.T.J., Olson P.D., Kinsella J.M., Swiderski Z. (2003). Molecular phylogenetic analysis of the Microphalloidea Ward, 1901 (Trematoda: Digenea). Syst. Parasitol..

[bib19] Tkach V.V., Pawlowski J. (1999). A new method of DNA extraction from the ethanol-fixed parasitic worms. Acta Parasitol..

[bib20] Trueb L., Massemin D. (2001). The osteology and relationships of *Pipa aspera* (Amphibia: Anura: Pipidae), with notes on its natural history in French Guiana. Amphibia-Reptilia.

[bib21] Van Steenkiste N., Locke S.A., Castelin M., Marcogliese D.J., Abbott C.L. (2015). New primers for DNA barcoding of digeneans and cestodes (Platyhelminthes). Mol. Ecol. Resour..

